# Single-Cell RNA-Sequencing Reveals the Active Involvement of Macrophage Polarizations in Pulmonary Hypertension

**DOI:** 10.1155/2022/5398157

**Published:** 2022-08-26

**Authors:** Xulong Mao, Yaozhe Li, Rui Yang, Jingqiu Wei, Zhucheng Zhao, Ting Zhang, Mingli OuYang, Xiaoling Liu, Can Liu, Hao Xu, Xiaoying Huang, Liangxing Wang

**Affiliations:** ^1^Key Laboratory of Heart and Lung, Division of Pulmonary Medicine, The First Affiliated Hospital of Wenzhou Medical University, Wenzhou, Zhejiang 325035, China; ^2^School of Ophthalmology & Optometry, School of Biomedical Engineering, Wenzhou Medical University, Wenzhou, Zhejiang 325035, China; ^3^Department of Rheumatology, The First Affiliated Hospital of Wenzhou Medical University, Wenzhou, Zhejiang 325035, China

## Abstract

**Background:**

Sustained hypoxia can trigger a progressive rise in pulmonary artery pressure and cause serious pulmonary diseases. Macrophages play important roles along the progression of pulmonary hypertension. However, the state of macrophage polarization during the early stage of pulmonary hypertension is unclear.

**Methods:**

Unlike traditional sequencing method, single-cell sequencing can accurately distinguish among cell types and better understand cell-to-cell relationships. In this study, we investigated the polarization of macrophages in pulmonary hypertension via single-cell RNA-sequencing in a mice hypoxia model, which was then validated in patients with pulmonary hypertension.

**Results:**

We identified that the intermittent exposure to hypoxic conditions could lead to the production of more M2-type macrophages than M1-type macrophages in a mouse model. Further validation analysis was performed by analyzing lung tissue of patients with pulmonary hypertension, revealing that the number of disease-associated M2 macrophages was substantially increased.

**Conclusions:**

In this study, the active anti-inflammatory response of macrophage involved in pulmonary hypertension has been identified, suggesting that intervention against the polarization of macrophages to the M2 type may be a potential way to reduce chronic pulmonary inflammation, pulmonary vascular remodeling, and artery pressure. Thus, investigation of macrophage polarization associated with hypoxia could help us better understand disease mechanism and craft effective prevention strategies and approaches.

## 1. Introduction

Maladaptive inflammatory signaling is a critical contributor to the progression of obliterative pulmonary arteriolar lesions and right ventricle (RV) failure in pulmonary arterial hypertension (PAH) [1, 2]. Although the exact pathophysiology of PAH is still unknown, there are growing evidences showing that inflammation may play an important role in the remodeling of the pulmonary arteries, leading to the development and progression of PAH. Hypoxia-induced pulmonary hypertension (PH) may be one of the most common reasons for cardiovascular disease, contributing to several pulmonary related diseases, such as sleep apnea, chronic obstructive pulmonary disease, and interstitial lung disease [3]. Meanwhile, several genetic and environmental factors (such as high altitude) play a predisposing or facilitating role in developing PAH [4]. Chronic hypoxia-induced PAH is a common type of PAH, which can cause respiratory function disorders such as chronic obstructive pulmonary disease (COPD) and obstructive sleep apnea [5]. The pulmonary hypoxic response is an evolutionarily conserved stress response that is triggered due to reduced alveolar oxygen availability [6]. Hypoxia induces an inflammatory response in the lungs and is characterized by alternative activation of macrophages with an elevation in the levels of proinflammatory mediators, which is critical for the development of hypoxic pulmonary hypertension (HPH) [7]. A hypoxic microenvironment triggers a metabolic adaptation in macrophages, increasing mitogenesis, invasion, angiogenesis, and similar biological processes [8].

Macrophages are a type of white blood cell of the immune system, which are mononuclear cells functioning as professional phagocytes, participating into many physiological processes, such as growth and development, homeostasis, damage repair, and preventing outside pathogens from invading human body. Changes in the external stimuli and microenvironmental conditions can induce macrophage polarization to different phenotypes, including the proinflammatory M1 phenotype and the anti-inflammatory M2 phenotype. The growing evidence suggests that chronic inflammation plays a central role in pulmonary vascular remodeling [9, 10]. However, the link between pulmonary vascular remodeling and PAH is still unclear. The role of macrophages in the progression of pulmonary hypertension has not yet been fully investigated.

This work aims to investigate the polarization status of macrophages responding to pulmonary hypertension. Using bioinformatics data obtained from single-cell RNA-sequencing, we identified a population of disease-associated macrophages in a mouse model with the intermittent hypoxia. The RNA-seq data of patients with pulmonary hypertension was obtained for validation. The effect of immune cell infiltration on occurrence of pulmonary hypertension and the involved hub genes related to immune cell invasion will be studied. Identifying new molecular targets is beneficial for the development of more effective drugs targeting PAH and related diseases. In this context, CX3CR1 and its target protein, a pleiotropic upstream proinflammatory mediator, are emerging as promising molecular targets. These molecules contribute to perivascular inflammation and pulmonary arterial remodeling, which are hallmarks of PAH that are not specifically targeted by currently approved therapies.

## 2. Methods

### 2.1. Data Source

The bioinformatics analysis was conducted using the public databases. These data were downloaded from the GSE145435 dataset of the Gene Expression Omnibus (GEO) database. The single-cell sequencing data (GSE145435) is consisted of 14 mice (7 control and 7 hypoxic mice), which were further divided among 3 separate experiments for discovering the role of macrophages in a mice hypoxic model. The RNA-seq data of patients with pulmonary hypertension was obtained from GSE113439, which contains 15 PAH patients and 11 healthy controls for validation.

### 2.2. Seurat Object Creation and Data Preprocessing

The “Read10×” function in the Seurat package was used to analyze the single-cell sequencing data derived from isolated murine lung cells. Seurat objects were created using the “CreateSeuratObject” function. The “FilterCells” function was used to filter the data and remove the effects of dead cells and cell adhesion. The specific parameters used in subsequent analyses are as follows: NFEATURE_RNA>1000, nFEATURE_RNA<2500, and PERCENT.mt<6, leaving 15,868 cells and 16,231 corresponding genes. Data were normalized using the “NormalizeData” function with the following parameters: normalize.method = ^“^LogNormalize^”^ and scale.factor = 10,000.

The high variation genes were calculated using the “FindVariableGenes” function, with the parameters set to default. The “ScaleData” function was further used to normalize the data and remove the sources of variation. The principal component analysis (PCA), Uniform Manifold Approximation and Projection (UMAP), and TSNE clustering results were obtained via analysis using the “BiocManager,” “gene set variation analysis (GSVA),” and “GSEABASE” packets in the R language, respectively.

### 2.3. Screening and Annotation of Cell Marker Genes in Different Subpopulations

The screening and annotations of different cell subpopulations were compared among all cell subpopulations and obtained genes per subgroup. The identification of each cell was based on the differential expression of characteristic genes among the various cell clusters. Subsequently, the cell tag gene was analyzed in the R language using the “CellMarker” website (http://biocc.hrbmu.edu.cn/CellMarker/), which determines the type of cells corresponding to each subpopulation.

### 2.4. Gene Ontology (GO) Annotation and Kyoto Encyclopedia of Genes and Genomes (KEGG) Pathway Enrichment Analyses

GO annotation and KEGG pathway enrichment analyses of the differentially expressed genes (DEGs) were performed using the Database for Annotation, Visualization, and Integrated Discovery (DAVID).The relevant terms were selected at an enrichment significance of *P* < 0.05. These analyses revealed the biological processes (BPs), cellular components (CCs), molecular functions (MFs), and metabolic pathways associated with the differentially expressed mRNAs.

### 2.5. Differential Expression Analysis of Mice Intermittently Exposed to Hypoxic Conditions

We analyzed single-cell sequencing data of lung tissue cells from mice exposed to intermittent hypoxia. Cells of cluster 5 were identified as M2-type macrophages via cell labeling analysis. The CX3CR1 gene is related to the polarization of M1 macrophages and the cluster 13 cells expressed high levels of CX3CR1 gene. We investigated the differential genes between cluster 5 and cluster 13 and conducted subsequent analyses to determine the genes related to the polarization of M1/M2 macrophages in the lung tissue exposed to intermittent hypoxia.

### 2.6. Hub Genes Analysis

The STRING database (http://string-db.org/) and Cytoscape software were used for joint analysis and to construct a macrophage marker gene-protein interaction network [11–16]. The hub genes were screened based on their connectivity using the MCODE plug-in.

### 2.7. GEO Bulk RNA-Seq Dataset Analysis to Validate the Hub Genes Related to Immune Cell Invasion

We selected the differential genes between the M2-type macrophage and the cell cluster expressing the polarization-related gene CX3CR1 of the M1-type macrophage cluster as the hub gene. The GSE113439 dataset obtained from patients with pulmonary hypertension was used for this analysis. The differential genes between the pulmonary hypertension group and the control group were determined, and then the hub genes were obtained from the differential genes. The expression matrix of the hub genes was then extracted. The level of immune cell invasion in the lung tissues of patients with pulmonary hypertension was calculated using the R package “CIBERSORT”.

## 3. Results

### 3.1. Identification of 16 Cell Clusters Based on scRNA-Seq Data

**Figure 1:**
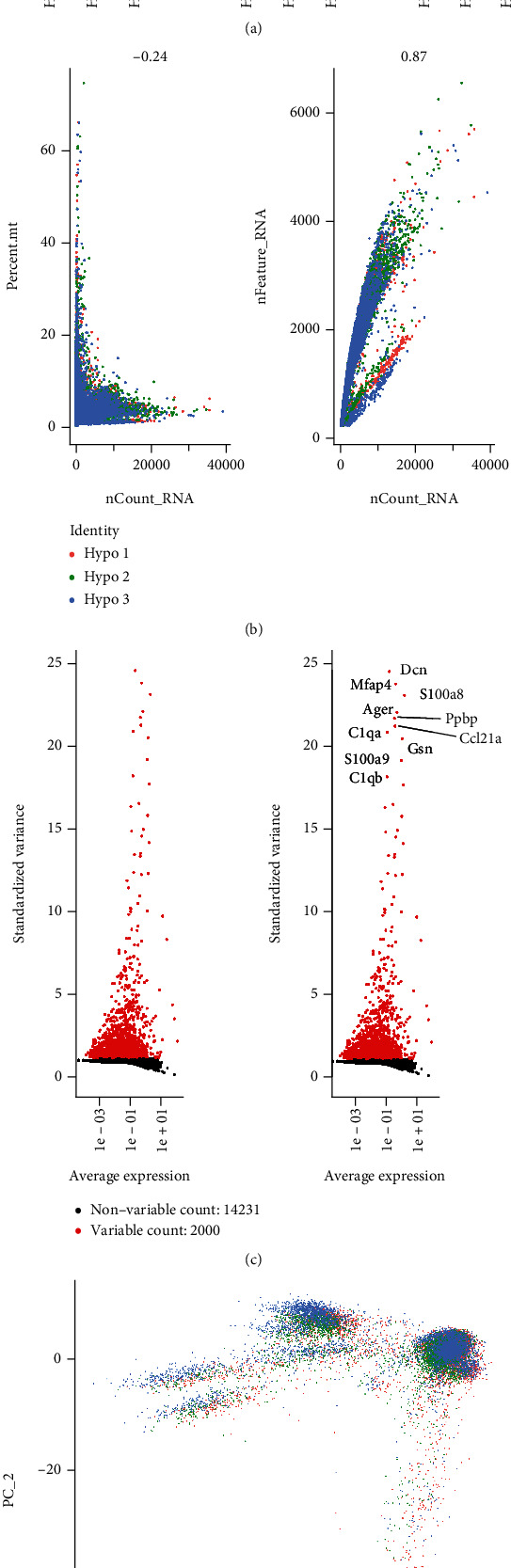
Characterization of single-cell sequencing from 24,965 cell samples from mice exposed to IH. (a) Quality control of single-cell RNA-seq for three subpopulations. (b) Correlation analysis between nCount RNA and nFeature RNA. (c) The subset of features that exhibit high cell-to-cell variation in dataset. Red dots mean 2,000 variable genes. The top ten gene names are pointed out. (d) Scatter plot showing the scores of individual cells (points) along the top two principal components.

**Figure 2:**
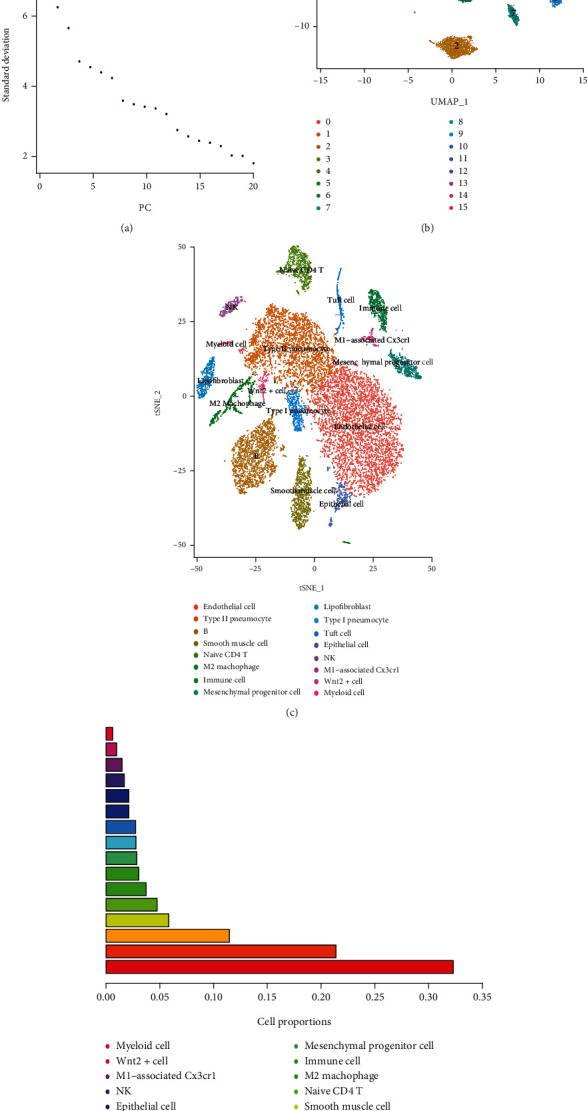
(a) Standard deviation (*y*-axis) accounted for top 20 PCs (*x*-axis) to identify the number of significant PCs based on the presence of an “elbow.” Approximately 15 PCS are chosen for the analysis. (b) Single cells onto 2-D UMAP space colored by assigned cell types. (c) Visualization of the data using t-distributed stochastic neighbor embedding (TSNE) after clustering based on transcriptomic similarity. (d) The proportion of cells in each cluster.

Following the quality control standards and the normalization of scRNA-seq data from hypoxia-exposed lung tissues of hypoxia-exposed mice, 15,868 cells from hypoxic cores were included in the final analysis. Low-quality cells were excluded from downstream analyses when they failed to meet the following criteria: (1) ≥50,000 sequence reads and (2) ≥40% of reads uniquely aligned to the genome. We applied “LogNormalize” to normalize the single-cell gene expression data and identified the highly variable genes using the function “FindVariableGenes” and the “vst” method (Figure 1(a)). The PCA of the single-cell expression matrix was performed to determine the significantly highly variable genes using the “RunPCA” function (Figure 1(b)). We then used the t-distributed stochastic neighbor embedding (TSNE) function to obtain cell scatterplots, and a total of 16,231 corresponding genes were included (2,000 variable features) (Figure 1(c)). The results demonstrated clear separations among cells in the hypoxia lung tissue (Figure 1(d)). The top eight most significantly correlated genes, both positively and negatively, are displayed as heatmaps in Supplementary Figure 1. The ScoreJackStraw were then performed to identify the available dimensions and screen the correlated genes (Figure 2(a)). Subsequently, we selected 15 principal components (PCs) with an estimated *P* value <0.05. Afterward, the TSNE algorithm was applied, and the cells from the mouse hypoxia lung tissue of mouse were successfully classified into 16 separate clusters (Figure 2(b)). The gene expression markers of the identity classes were acquired using the “FindMarkers” function (min.pct = 0.25, logFC threshold = 0.25). According to the expression patterns of the marker genes, the clusters were annotated using CellMarker (Figure 2(c)). The proportion of cells in cluster 0 to 15 was descending. Specifically, the cluster 5 containing 933 cells was annotated as typical M2 macrophages, and the cluster 13 containing 327 cells were annotated as CX3CR1-positive cells expressing CX3CR1, where CX3CR1 was associated with typical M1 macrophages (Figure 2(d)).

### 3.2. Identification of DEGs

**Figure 3:**
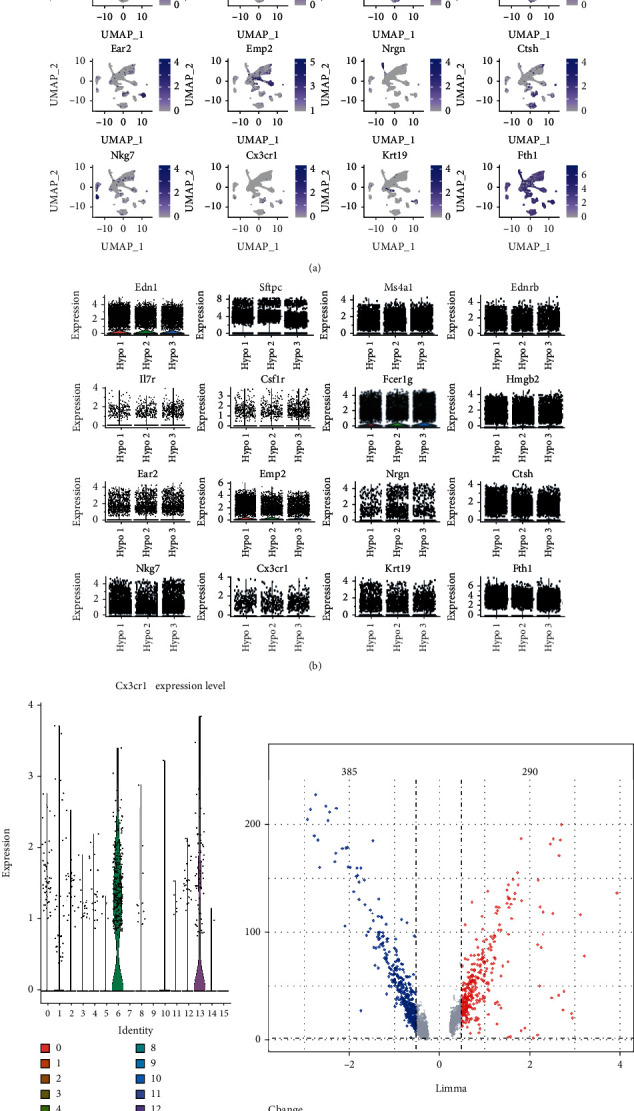
(a) The sixteen classic markers known to distinguish M1-type macrophage from the other cells. (b) The top sixteen differential genes between the cluster 5 cells (M2-type macrophage) and cluster 13 cells (T cells expressing the CX3CR1 gene associated with M1-type macrophages). (c) The CX3CR1 was mainly expressed in cluster 13. (d) Volcano plot of differentially expressed genes between cluster 5 and cluster 13. Red and blue indicate higher expression and lower expression, respectively.

Sixteen classic markers have been identified to distinguish M1-type macrophages from the other cells, which are shown in purple on UMAP by cell marker genes (Figure 3(a)). The expression levels of the 16 differentially genes among the clusters in the three hypoxia samples are shown in Figure 3(b), and the expression levels of CX3CR1 gene in cluster 13 cells are shown in Figure 3(c). Following data normalization, a differential gene expression analysis was performed between the cells in cluster 5 (M2 macrophages) and cluster 13 (highly associated with M1 macrophages) obtained from the lung tissue of mice exposed to intermittent hypoxia. A total of 675 DEGs were screened based on the standard parameters of FDR<0.05 and log2 FC>1. Among these DEGs, 290 genes were upregulated (indicated as red dots), and 385 genes were downregulated (blue dots) as shown in Figure 3(d).

### 3.3. GO Term and KEGG Pathway Enrichment Analysis of Screened DEGs

**Figure 4:**
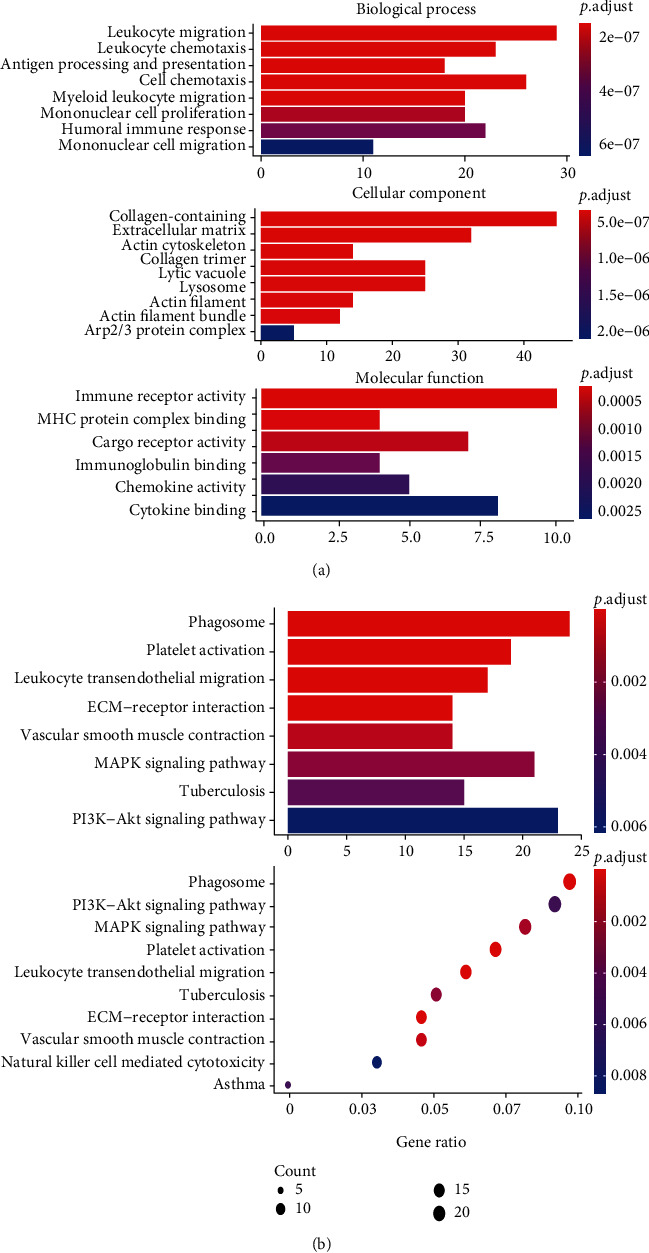
Functional enrichment analysis of the genes. The *x*-axis shows the ratio number of genes, and the *y*-axis shows the pathway terms. The -log_10_(*P* value) of each term is colored according to the legend. (a) Gene ontology analysis. (b) KEGG pathway analysis.

To further investigate the underlying functions of the DEGs between cluster 5 cells and cluster 13 cells, enrichment analysis was conducted. Results showed 675 marker genes which were further analyzed to determine the gene symbols and their potential functions. GO functional analysis showed that the DEGs were involved in 416 biological processes, 55 cellular components, and 48 molecular functions, with an adjusted *P* < 0.05. (Table 1). Noteworthily, the screened DEGs were especially enriched in the GO terms of myeloid leukocyte migration, leukocyte migration, leukocyte chemotaxis, immune receptor activity, extracellular matrix, and cytokine binding. Further analyses showed that these GO items were potentially involved in the progression of inflammation resulting from a brief lack of oxygen in lung tissue (Figure 4(a)). The KEGG analysis results revealed the enriched pathways mainly in the phagosome, ECM-receptor interactions, leukocyte transendothelial migration, vascular smooth muscle contraction, and P13K-Akt signaling pathway (Figure 4(b)).

### 3.4. Gene Set Enrichment Analysis (GSEA)

**Figure 5:**
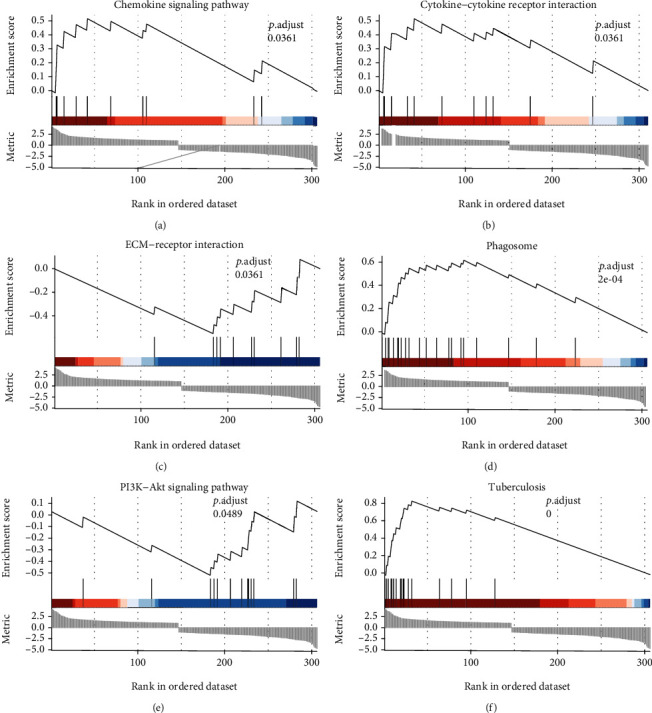
GSEA showing the differential gens play a key role in (a) chemokine signaling pathway, (b) cytokine-cytokine receptor interaction, (c) ECM-receptor interaction, (d) phagosome, (e) P13K-Akt signaling pathway, and (f) tuberculosis.

The gene set enrichment analysis was performed using the GSEA software (https://www.broadinstitute.org/gsea/). GSEA results revealed the association of the chemokine signaling pathway, cytokine-cytokine receptor interaction, ECM-receptor interaction, phagosome, PI3K-Akt signaling pathway, and tuberculosis (Figures 5(a)–5(f)). These pathways have a definite influence on the occurrence and development of inflammation and pulmonary hypertension. The chemokine signaling pathway may be an important pathway leading to the development of pulmonary hypertension. Previous studies have also reported that CX3CR1 plays an important role in the polarization of macrophages [17], which is essential in the development of pulmonary hypertension.

### 3.5. Hub Genes Analysis

We used the STRING database to construct a protein-protein interaction (PPI) network containing the selected differentially expressed genes. When the cutoff was set as a combined score>0.7, a total of 5,760 protein pairs and 893 nodes were included in the network. We then constructed and visualized the PPI network of DEGs based on the results of the STRING database analysis. In total, 864 nodes (genes) and 3,878 edges (interactions) were screened. We next identified the emerging modules from the PPI network using the MCODE plug-in, and then the top three central modules with MCODE scores >10 were selected (Figure S2). Specifically, module 1 had an MCODE score of 77.063 and consisted of 80 nodes and 3,044 edges (Figure S2A); module 2 had an MCODE score of 21.6 and consisted of 41 nodes and 432 edges (Figure S2B); and module 3 had an MCODE score of 15,729 and consisted of 60 nodes and 464 edges (Figure S2C). Through GO and KEGG pathway enrichment analyses, module 1 was found to be enriched mainly in biological processes, and module 2 was enriched mainly in endocytosis and positive regulation of MAPK cascade. Module 3 was enriched mainly in chemokine signaling pathway, regulation of type I hypersensitivity, T cell chemotaxis, lymphocyte chemotaxis, and leukocyte chemotaxis (Table 1).

In the established PPI network, CX3CR1 and its interacting genes were also identified as hub genes. The other DEGs included *Ccl5* (C-C motif chemokine 5), *C3ar1* (C3a anaphylatoxin chemotactic receptor), *Psap* (prosaposin), *Gng11* (guanine nucleotide-binding protein G(I)/G(S)/G(O) subunit gamma-11), *Ccl9* (C-C motif chemokine 9), *Pf4* (platelet factor 4), *Ccr2* (C-C chemokine receptor type 2), *Ccl6* (C-C motif chemokine 6), *Gpr183* (G-protein-coupled receptor 183), and *Cxcl16* (C-X-C motif chemokine 16) and were all included in module 3 (Table 2).

### 3.6. Validation of Immune Cell Invasion during Pulmonary Hypertension

**Figure 6:**
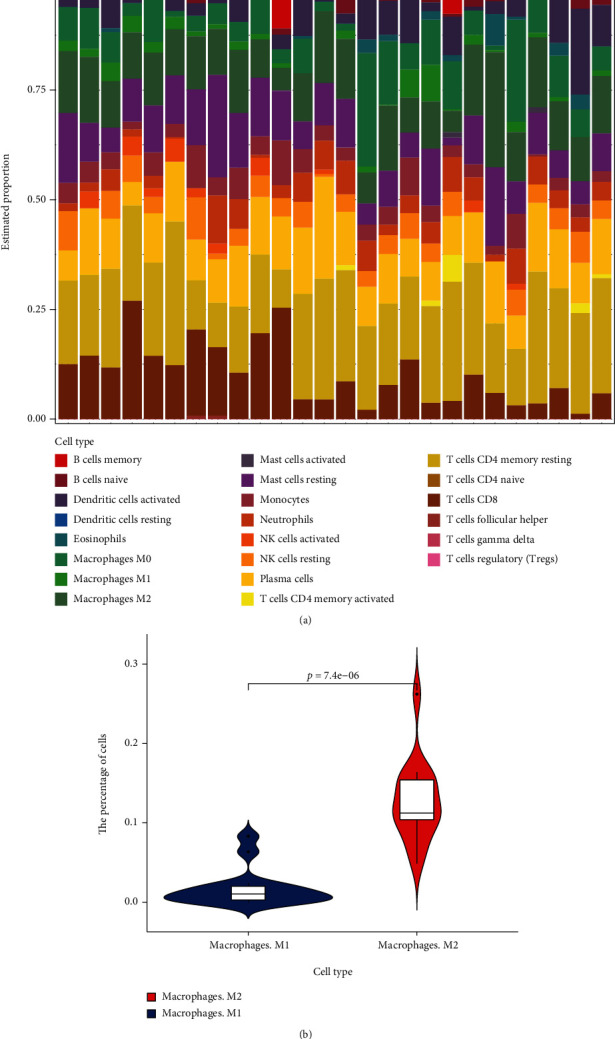
(a) Estimation of fractions of 22 immune cells using the CIBERSORT, and the 22 immunes cells were annotated by different colors. (b) Compared the percentage of macrophages M1 and M2 in PAH groups (*P* < = 7.4e^−06^).

Given the important role of infiltrating immune cells during pulmonary hypertension progression, we integrated a comprehensive analysis of immune signatures with the identity of the immune infiltrates. We extracted the expression matrices of DEGs (identified from the single-cell RNA-seq) from the GSE113439 microarray dataset in the GEO database and then calculated the extent of immune cell invasion in the lung tissue of patients with pulmonary hypertension according to the CIBERSORT formula. 15 pulmonary hypertension samples and 11 control samples were applied for CIBERSORT analysis. The immunocyte subpopulations of each sample are summarized, and the percentages and subpopulations of the infiltrating immune cells are presented in Figure 6(a). In addition, compared with the control, a higher proportion of plasma cells, eosinophils, and dendritic cells activated were detected in the pulmonary hypertension samples. On the contrary, lower proportions of resting memory CD4 T cells, regulatory T cells (Tregs), activated NK cells, M0 macrophages, M1 macrophages and M2 macrophages, and resting mast cells were observed in the samples from patients with pulmonary hypertension, compared to the normal samples. We further investigated the proportion of macrophage M1 and M2, which reveals that the number of M2 type is much higher than macrophage M1 in PAH patients (Figure 6(b)). M2-activated macrophages are closely related to tissue repair and regeneration and might participate into vascular remodeling in PAH. Thus, the elevated level of M2 macrophage indicates the active participation of macrophage polarization during progression of PAH.

## 4. Discussion

Our results showed that the expression of *CX3CR1*, *CCL5*, *C3AR1*, *PSAP*, *GNG11*, *PF4*, *CCR2*, *GPR183*, and *CXCL16* were associated with the levels of immune cell infiltration in the lung tissue of mice exposed to hypoxia. Further, M2 macrophages, but not M1 macrophages, may be involved in the pathophysiology of inflammation during the early stages of pulmonary hypertension. Based on the analysis of single-cell sequencing data, we found that hypoxia caused the polarization of macrophages to the M2 phenotype, leading to an inflammatory response.

Several cytokines (CX3CR1, CCL5, C3AR1, PSAP, GNG11, PF4, CCR2, GPR183, and CXCL16) are involved in the polarization of macrophages or the inflammatory response and may also be associated with the inflammatory regulation of pulmonary hypertension. The interaction between CX3CL1 and CX3CR1 may serve as a compensatory mechanism to preserve or augment the proinflammatory process of intercellular interactions [17]. Upregulation of the hypoxia response was related to an increased expression of CX3CR1, a lymphocyte survival factor. It leads to an increase in the number of inflammatory cells infiltrating into the lung tissue [18, 19]. The expression of CX3CR1 may also contribute to creating a microenvironment in the lungs that promotes macrophage polarization to the M1 phenotype. Several lines of evidence support that hypoxia strongly affects macrophage functions and indicate that the immune response and energy metabolism are interrelated events [8]. In the early stages of pulmonary hypertension, chronic exposure to intermittent hypoxia may lead to selective activation of inflammatory response pathways [20].

Further, mRNA expression analysis in blood macrophages identified six markers of M1 polarization (IL-12p35, CXCL10, CXCL11, CCL5, CCR7, and IDO1) and five markers of M2 polarization (TGF-*β*, CCL14, CCL22, SR-B1, and PPAR*γ*) [21]. The key chemokine pathways involved were associated with macrophage recruitment and pulmonary vascular remodeling, such as the CCL5-CCR5 pathway [22, 23]. Interestingly, in mice exposed to hypoxia or SUGEN-induced pulmonary hypertension, targeting both CCR2 and CCR5 prevented or reversed PH more efficiently than targeting either receptor alone [24]. Intravenous injection with human rPF4 protein into lambs induced acute pulmonary vasoconstriction and hypertension associated with thromboxane release in the circulating blood [14]. A previous study reported that CXCL16 was elevated in the systemic sclerosis (SSc) serum from the patients with pulmonary arterial hypertension [16]. The hypoxia can strongly induce the expressions of CXCR6, which is a receptor for CXCL16 via hypoxia-inducible factor-1*α* [15]. This suggests that these chemokines may play an important role in progressions of pulmonary arterial hypertension [25].

C3AR1 mRNA expression was positively correlated to the infiltration levels of various immune cells such as macrophages [26]. EBI2 expression directs the date of Tfh cells by promoting their interaction with IL-2-quenching dendritic cells. Moreover, the EBI2 signaling pathway appears to be a sensor of immune challenge. For example, exposure to lipopolysaccharides promoted EBI2 signaling in B cells, macrophages, and other immune cells, upregulating the expression of EBI2 (also known as GPR183), CYP7B1, and CH25H while downregulating HSD3B7 expression [27]. A previous study reported that plasma CXCL16 concentrations were also significantly increased in patients with IPAH (idiopathic pulmonary hypertension) compared with the control subjects [19].

Intrapulmonary arteries usually constrict due to alveolar hypoxia. PAH is considered a fatal disease with its molecular mechanism still unknown. In ovarian cancer cells, CX3CR1 was upregulated in a course of hypoxia-mediated regulation of hypoxia-inducible factor-1*α* (HIF-1*α*) [18]. Hypoxia, a common phenomenon in malignant tumors, speeds up cell proliferation regulated by HIF-1*α* expression. Hung et al. in 2007 demonstrated for the first time that the upregulation of CX3R1 is dependent on HIF-1*α* expression [28]. Sustained hypoxia activates Rho kinase, inducing vasoconstriction and the expression of HIF-1*α*, leading to adverse pulmonary vascular remodeling and eventually PH [29].

These findings also emphasize the complexity of the role inflammatory cells may play during vascular remodeling in pulmonary tissues. Although several studies have investigated the role of specific chemokines and chemokine receptors in the development of PH, of particular interest are the potential interactions between CX3CR1 and macrophages, both of which are involved in monocyte/macrophage lineage recruitment and affect PA-smooth muscle cell (SMC) functions. Therefore, the present study aimed to elucidate the individual and combined actions of these systems in mice with PH induced by exposure to hypoxia.

A model of pulmonary arterial hypertension can be established via continuous hypoxia in mice. This study found a significant enrichment of DEGs related to myeloid leukocyte migration and cytokine binding associated with hypoxia-induced inflammatory processes through the proliferation of vascular smooth muscle cells. M1-type macrophage marker genes were obtained via single-cell bioinformatics data from processing hypoxia-exposed lung tissue, and the biological processes where these marker genes were enriched were elucidated. We also explored the potential cellular mechanisms of these marker genes and DEGs through gene set enrichment analysis (GSEA). The chemokine signaling pathway and the PI3K-Akt signaling pathway, as obtained from the GSEA analysis, may be involved in the inflammatory response in the absence of an external trigger. Vascular smooth muscle contraction and metabolism of xenobiotics by cytochrome P450 may also contribute to the progress of pulmonary hypertension. Voelkel NF demonstrated that the cytochrome P450 system (activated by endogenous and exogenous AhR ligands) might control hypoxia, inflammation, angiogenesis, and maybe even quasi-malignant growth, which are hallmarks of the pathobiology of PAH [30, 31]. Although largely distinct and seemingly unrelated, asthma and PAH have important pathological features in common, including hypoxia, inflammation, smooth muscle contraction, and vascular remodeling [32]. Our integrated analyses identified the key pathways in patients with PAH, including cAMP, ECM-receptor interaction, AMPK, hypoxia-inducible factor 1-*α*, and the PI3K-Akt signaling pathways [33]. The activated PI3K-Akt signaling pathways can promote the proliferation of ligamentum flavum cells [34]; mechanistically, it may lead to the proliferation of pulmonary smooth cells in PAH.

Persistent hypoxia can cause pulmonary hypertension, so intermittent exposure to hypoxic conditions can be considered as an early risk factor for pulmonary hypertension. Following intermittent exposure to hypoxia, mice showed an increased number of M2-type macrophages than M1-type macrophages. Elucidation of the molecular pathways underlying macrophage adaptation to hypoxia is expected to provide novel therapeutic strategies for PAH. Our analyses from the single-cell sequencing of mouse lung tissue after exposure to hypoxia confirmed that M2-type macrophages, but not M1-type macrophages, were the dominant cells in these tissues. A continuous lack of oxygen can cause many diseases in the lungs, such as pulmonary hypertension. We also validated the presence of immune cell invasion in patients with pulmonary hypertension using a pulmonary arterial hypertension dataset obtained from a publicly available database. M2-type macrophages dominated over M1-type macrophages among the 22 kinds of immune cells obtained via bioinformatics analysis of the dataset. Therefore, M2-type macrophages may be the cells that primarily cause inflammation in patients with pulmonary hypertension. Active intervention against the polarization of macrophages to the M2 type rather than the M1 type may reduce pulmonary inflammation, improve pulmonary vascular remodeling, and reduce pulmonary artery pressure. This intervention may pave the way to developing therapeutic modalities for pulmonary hypertension from the perspective of inflammation.

## 5. Conclusions

Macrophages may serve as very important targets for treating pulmonary hypertension. More and more evidence indicate that M2-type macrophages can cause chronic inflammation and pulmonary vascular remodeling in patients with pulmonary hypertension. In this work, we evaluate the polarization status of macrophage via single-cell RNA-sequencing, revealing that the proportion of the disease-associated M2 macrophages substantially increased in progression of PAH. Our study suggests that the inhibition of CX3CR1 expression could be a potential strategy for hypoxia-induced lung disease treatment, and also, to some extent, the activation of innate immunity such as macrophage polarization might be an effective therapeutic target towards PAH disease. For future directions, it will be more guiding significance to further confirm the functional role of macrophages and other immune cells in lung tissues of patients with pulmonary hypertension. Also, the molecular mechanism and underlying etiology regarding the relationships between macrophage polarization and PAH development are expected to be deeply explored.

## Figures and Tables

**Table 1:** The enriched GO and KEGG pathways of DEGs in the top three modules.

Cluster	Term	Description	Count	False discovery rate
Module 1				
Go:BP	GO:0030100	Regulation of endocytosis	15	0.00060
Go:BP	GO:0060326	Cell chemotaxis	9	
Go:CC	GO:0098772	Molecular function regulator	25	0.00060
Module 2				
KEGG	mmu04144	Endocytosis	12	5.28e-06
Go:BP	GO:0043410	Positive regulation of MAPK cascade	5	8.45e-06
KEGG	mmu03320	PPAR signaling pathway	3	2.63e-05
KEGG	GO:0032088	Negative regulation of NF-kappaB transcription factor activity	3	2.63e-05
Module 3				
Go:BP	GO:0001810	Regulation of type I hypersensitivity	3	1.10e-09
Go:BP	GO:0010818	T cell chemotaxis	3	0.0073
Go:BP	GO:0048247	Lymphocyte chemotaxis	9	1.58e-05
Go:BP	GO:0048247	Lymphocyte chemotaxis	9	<0.0001
Go:BP	GO:0030595	Leukocyte chemotaxis	12	<0.001
Go:BP	GO:0070098	Chemokine-mediated signaling pathway	6	8.80e-07
Go:MF	GO:0048020	CCR chemokine receptor binding	4	6.76e-05
KEGG	mmu04062	Chemokine signaling pathway	13	4.26e-13

**Table 2:** The hub genes identified by the MCODE plug-in.

Gene symbol	Description	Degree of connectivity	logFC
CX3CR1	The chemokine (C-X3-C) motif receptor 1	24	1.40
Ccl5	C-C motif chemokine5	22	1.13
C3ar1	C3a anaphylatoxin chemotactic receptor	42	1.29
Psap	Prosaposin	33	1.20
Gng11	Guanine nucleotide-binding protein G(I)/G(S)/G(O) subunit gamma-11	26	-1.9
Ccl9	C-C motif chemokine 9	20	3.42
Pf4	Platelet factor 4	27	2.48
Ccr2	C-C chemokine receptor type 2	25	1.90
Ccl6	C-C motif chemokine 6	22	3.62
Gpr183	G-protein coupled receptor 183	17	1.09
Cxcl16	C-X-C motif chemokine 16	22	1.15

## Data Availability

The RNA-seq data including single-cell RNA-seq data of the mice model for discovering and normal RNA-seq data of the PH patients for validation were obtained from public resources with the accession number GSE145435 (https://www.ncbi.nlm.nih.gov/geo/query/acc.cgi?acc=GSE145435) and GSE113439 (https://www.ncbi.nlm.nih.gov/geo/query/acc.cgi?acc=GSE113439), respectively.

## Abbreviations

BP: Biological process
CC: Cellular component
COPD: Chronic obstructive pulmonary disease
DAVID: Database for Annotation, Visualization, and Integrated Discovery
DEG: Differentially expressed gene
GEO: Gene Expression Omnibus
GO: Gene ontology
GSEA: Gene set enrichment analysis
GSVA: Gene set variation analysis
HPH: Hypoxic pulmonary hypertension
KEGG: Kyoto Encyclopedia of Genes and Genomes
HIF-1*α*: Hypoxia-inducible factor-1*α*
MF: Molecular function
PAH: Pulmonary arterial hypertension
PCA: Principal component analysis
pH: Pulmonary hypertension
PPI: Protein-protein interaction
RV: Right ventricle
TSNE: T-distributed stochastic neighbor embedding
SMC: Smooth muscle cell
UMAP: Uniform Manifold Approximation and Projection.

## Supplementary Materials

Supplementary Materials Figure S1: heatmaps showing expression of top 8 positive and negative loading in each cluster cells along PC-1 to PC-15. Figure S1: module analysis of the PPI network. The top three central modules of the PPI network were identified and visualized by using the MCODE plug-in in Cytoscape. (A) Module 1. (B) Module 2. (C) Module 3. The size of nodes from small to large indicates the degree of connectivity of nodes from low to high, and the hub genes are marked in yellow.
