# Supplementary material for: Single-Cell RNA-Sequencing Reveals the Active Involvement of Macrophage Polarizations in Pulmonary Hypertension
Source: Dis Markers. 2022 Aug 26;2022:5398157. doi: 10.1155/2022/5398157 (PMC9553540; doi:10.1155/2022/5398157)
Supplement: Supplementary Materials — Figure S1: heatmaps showing expression of top 8 positive and negative loading in each cluster cells along PC-1 to PC-15. Figure S1: module analysis of the PPI network. The top three central modules of the PPI network were identified and visualized by using the MCODE plug-in in Cytoscape. (A) Module 1. (B) Module 2. (C) Module 3. The size of nodes from small to large indicates the degree of connectivity of nodes from low to high, and the hub genes are marked in yellow. [file 5398157.f1.docx]

**Supplementary Information**

**Single-cell RNA-sequencing reveals the active involvement of macrophage polarizations in pulmonary hypertension**

Xulong Mao^1^_,_ Yaozhe Li^1^, Rui Yang^3^, Jingqiu Wei^1^, Zhucheng Zhao^1^, Ting Zhang^2^, Mingli OuYang^1^, Xiaoling Liu^3^, Can Liu^1^, Hao Xu^3^, Xiaoying Huang^1*^, Liangxing Wang^1*^

1. Key Laboratory of Heart and Lung, Division of Pulmonary Medicine, The First Affiliated Hospital of Wenzhou Medical University, Wenzhou, Zhejiang 325035, China.
2. Department of Rheumatology, The First Affiliated Hospital of Wenzhou Medical University, Wenzhou, Zhejiang, Zhejiang 325035, China.
3. School of Ophthalmology & Optometry, School of Biomedical Engineering, Wenzhou Medical University, Wenzhou, Zhejiang 325035, China.

*Corresponding authors: Xiaoying Huang (huangxiaoying@wzhospital.cn) and Liangxin Wang (wangliangxing@wzhospital.cn)


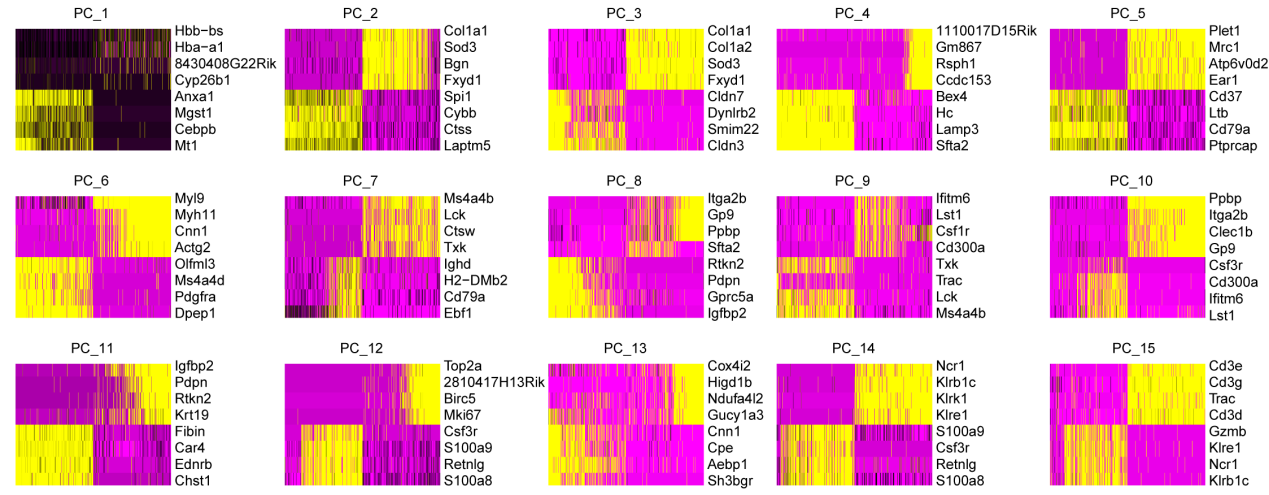


**FIGURE S1** Heatmaps showing expression of top 8 positive and negative loading in each cluster cells along PC-1 to PC-15.


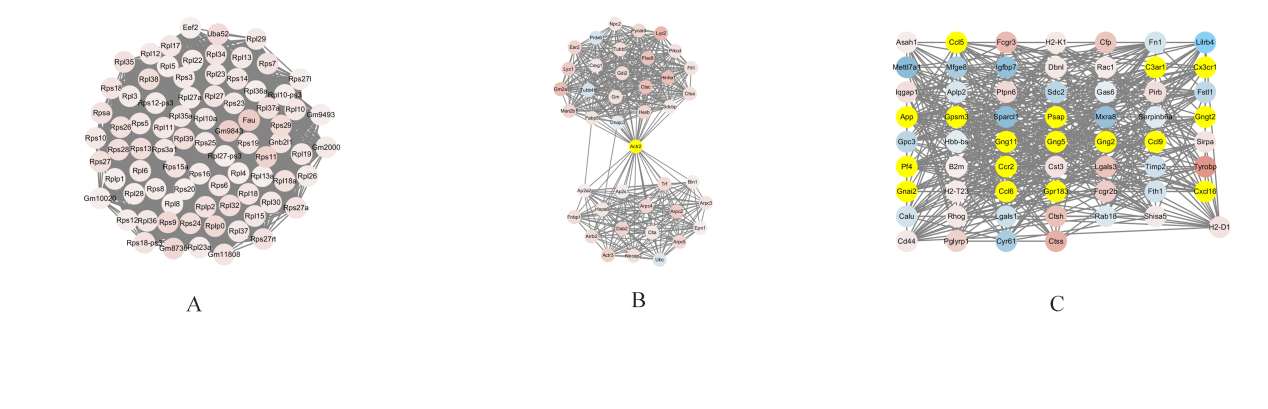


**FIGURE S2** Module analysis of the PPI network. The top three central modules of the PPI network were identified and visualized by using the MCODE plug-in in Cytoscape. (A) Module 1. (B) Module 2. (C) Module 3. The size of nodes from small to large indicates the degree of connectivity of nodes from low to high, and the hub genes are marked in yellow.
